# Loading of fish oil into β-cyclodextrin nanocomplexes for the production of a functional yogurt

**DOI:** 10.1016/j.fochx.2022.100406

**Published:** 2022-08-04

**Authors:** Tahere Ghorbanzade, Sahar Akhavan-Mahdavi, Mohammad Saeed Kharazmi, Salam A. Ibrahim, Seid Mahdi Jafari

**Affiliations:** a Department of Food Materials and Process Design Engineering, Gorgan University of Agricultural Sciences and Natural Resources, Gorgan, Iran; b Faculty of Medicine, University of California, Riverside, USA; c Food and Nutritional Sciences Program, North Carolina Agricultural and Technical State University, E. Market Street, 1601, Greensboro, NC 24711, USA; d Department of Analytical Chemistry and Food Science, Nutrition and Bromatology Group, Faculty of Science, Universidade de Vigo, E-32004 Ourense, Spain; e College of Food Science and Technology, Hebei Agricultural University, Baoding 071001, China

**Keywords:** Fortification, DHA, Fish oil, Encapsulation, Nanodelivery systems

## Abstract

Omega-3 fatty acids play a role in achieving optimal health and in protection against diseases. Although instability and oxidation of its essential fatty acids has limited its use in food products. Among the strategies used to prevent these challenges, the encapsulation technique has been the most successful method. Therefore, in this study, β-cyclodextrin (BCD) inclusion complexes were applied for encapsulation of fish oil and its addition into yogurt for fortification. Physicochemical properties of produced yogurt as well as sensory tests were investigated during 21 days of storage at 4 °C. The results showed that encapsulation of fish oil with BCD significantly reduced the acidity, peroxide value, and syneresis of yogurt while increasing docosahexaenoic acid (DHA) and eicosapentaenoic acid (EPA). In conclusion, the results demonstrated that yoghurt fortified with encapsulated fish oil has similar sensory qualities to the control sample than yoghurt fortified with free fish oil.

## Highlights

• The main limitation of adding fish oil into food products is its instability and oxidation.
• It leads to the production of improper aroma, unpleasant odor/taste of final product.
• β-cyclodextrin (BCD) inclusion complexes were applied for encapsulation of fish oil.
• Physicochemical properties of produced yogurt were investigated during storage at 4 °C.
• Adding encapsulated fish oil into yogurt gave closer properties to control sample.

## Graphical abstract

**Figure:**
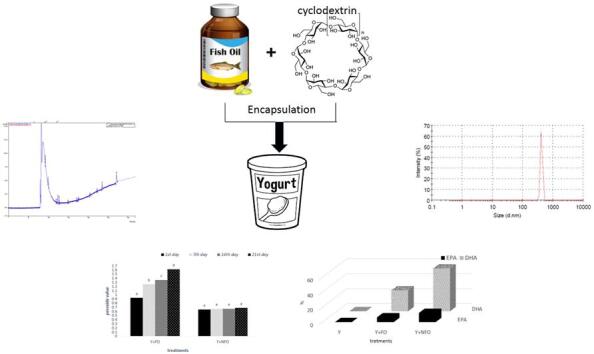

## Introduction

In recent years, oils having high concentrations of polyunsaturated fatty acids (PUFAs) are increasingly being used in food manufacturing. Studies have revealed that PUFAs protect us not just against cardiovascular disease, but also from mortality. (Shegelman et al., 2019). Fish oil is often considered as a source of omega-3 (ω − 3) fatty acids in the diet. Eicosapentaenoic acid (EPA) and Docosahexaenoic acid (DHA) are the most important PUFAs known as their important health benefits and positive effects in reducing cardiovascular diseases and preventing some cancers and inflammatory diseases of the immune system. Enriching various foods with fish oil is a method for increasing the consumption of fish oil. However, the biggest technological barrier to incorporating effective amounts of EPA and DHA into food is the occurring oxidation and odor of fish due to lipid breakdown, which must be avoided. (Feizollahi et al., 2018, Gumus and Gharibzahedi, 2021).

Various approaches to preventing lipid oxidation in omega-enriched foods have been studied, and encapsulation is suggested as the most successful method (Gowda et al., 2018, Muñoz-Tébar et al., 2019, Göksen et al., 2020). Encapsulation is a technique to entrap bioactive agents within a wall or carrier material and it is a valuable process to enhance delivery of bioactive molecules as well as their controlled release. Encapsulation of fish oil may create a protective barrier against oxidation, which in turn masks the unpleasant flavor of fish oil. The type of encapsulating agents or wall material used, as well as the encapsulation method, are critical factors to consider when assessing encapsulation. (Mahdavi et al., 2022).

Cyclodextrins are a family of cyclic oligosaccharides consisting of a macrocyclic ring containing glucose subunits that are linked by alpha-4.1 glycoside bonds that are produced through starch by enzymatic conversion (Liu, Chen, Gao, Fu, & Hu, 2020). The three main types of this group of compounds are α-cyclodextrin(6 glucose subunits), β-cyclodextrin(7 glucose subunits) and γ-cyclodextrin(8 glucose subunits). Cyclodextrins, particularly beta cyclodextrins, are frequently utilised in pharmacy because of their potential to improve drug solubility and stability by forming solid complexes. β-Cyclodextrin (BCDs) is a cone-shaped molecule with nanoscale cavities and is a collection of oligosaccharides that is hydrophilic on the outer surface due to many hydroxyl groups but is hydrophobic in the cavity (Kfoury, Auezova, Greige-Gerges, & Fourmentin, 2019). Consequently, BCD is soluble in water and various types of hydrophobic guest molecules can be encapsulated in its non-polar cavity and such a feature is widely used in the context of drug-controlled release, separation, and absorption. Compared to other forms of cyclodextrin, BCDs are more popular due to their low cost, better compatibility with the human body, and simpler reactions. (dos Santos, Buera, & Mazzobre, 2017). BCDs are important applications in the pharmaceutical, food, and nutraceutical industries to stabilize light or oxygen-sensitive substances, increase the chemical activity of the nucleus, stabilise volatiles, improve the solubility of hydrophobic substances with low solubility in water, protection of products against microorganisms, masking undesirable odor, and disguising the color of materials.

Fortification of food products with fish oil by encapsulation technique can be a way to increase its availability and improve consumer health. Various studies reported that used encapsulated fish oil and omega-3 fatty acids in food products such as bread (Ojagh and Hasani, 2018, Sridhar et al., 2021), chicken nuggets (Pourashouri, Shabanpour, Heydari, & Raeisi, 2021), sausage (Pourashouri, Shabanpour, Kordjazi, & Jamshidi, 2020), mayonnaise (Miguel et al., 2019), juice (Sridhar, Sharma, Choudhary, Dikkala, & Narsaiah, 2021), and snacks (Álvarez and Lamas 2021). Dairy products are good candidates for omega-3 fortification because of their high amount of consumption and stored under refrigerated conditions (Zhong et al., 2018, Hamed et al., 2019, Mieehsanpazir and Asadi 2021, Zakipour Rahimabadi 2021). In our previous work, we produced successfully nanoencapsulated fish oil by liposomes (Ghorbanzade, Jafari, Akhavan, & Hadavi, 2017). Since we found that there have not been any studies on the nanoencapsulation of fish oil using BCDs, therefore, the aim of this study was to investigate the nanoencapsulation of fish oil by BCDs and its addition to yogurt to produce a functional product; finally, to evaluate the different properties of fortified yogurt during storage time.

## Materials and methods

BCD (Cycloheptaamylose, C_42_H_70_O_35_ with a molecular weight of 1134.98) was obtained from Sigma Aldrich (St. Louis, USA). Yogurt, (2.8 % fat, Pegah, Iran) was bought from a local market. While, lecithin (Merck, Germany), purified fish oil (Jiangyin Shuji International Trade Co., China), sunflower oils (Nina, Iran), and all other chemicals as analytical grades and were purchased from reputable chemical suppliers.

### Encapsulation within β-cyclodextrin

BCD as a wall material (50 g) was dissolved in 500 mL of an ethanol to water (1:2) mixture maintained at ambient temperature for 24 h to ensure saturation of biopolymer molecules. Then 100 mL of fish oil (core material) was added to BCD mixture. The final mixture was stirred for 4 h at 24 °C for 2800 g to form an emulsion with a rotor–stator homogenizer (Ultra-Turrax, IKA, Germany). Finally, an ultrasonic homogenizer (Hielscher UP200Ht, Germany) equipped with a 14 mm thick titanium probe was used for encapsulation. The encapsulation process was performed by applying a power of 150 W and a frequency of 20 kHz at ambient temperature for 5 min (Karimi Sani, Alizadeh et al., 2019).

### Determining the efficiency of encapsulation

The amount of encapsulated fish oil in BCD nanocomplexes was estimated according to Erfani et al. (2019) with some modifications. To measure encapsulation efficiency, 1 mL of each sample was first centrifuged at 18,928 g for 30 min to separate the capsules. Then, in order to completely remove unseparated particles, the samples were filtered by a 0.22 μm injector filter. 40 μl of each sample was taken and diluted to 2 mL using methanol, and finally, the absorbance was read by spectrophotometer (PG-Instrument-ltd, UK) at a wavelength of 280 nm. The total fish oil content was calculated using the linear equation obtained from the calibration curve. Encapsulation efficiency (%EE) was calculated according to Eq.1 (Erfani, Pirouzifard, Almasi, & Gheybi, 2019).

(1) $$\%\;EE=\frac{amount\;of\;fish\;oil\;entrapped}{initial\;fish\;oil\;amount}\times 100$$

### Particle size distribution

The size distribution of BCD nanocomplexes was determined by a light scattering instrument (ZetaSizer, Malvern instruments ltd., Worcestershire, UK). Measurements were made using aqueous diluted samples (2:1 ratio). Basing on the principle of photon correlation spectrometry, this instrument also provides the possibility of the measurement of particle-size distributions in the range.

### Yogurt preparation

Pasteurized yogurt using 2.8 % fat was used to prepare the fortified yogurt formulation. Then 10 mL of BCD emulsion (containing 1 g of DHA + EPA) was added separately to the 100 g yogurt samples under stirring in yogurt until its full incorporation, without phase separation or precipitation. Then it stored at 4 °C in a refrigerator. The physicochemical properties of yogurts stored for 21 days of storage at 4 °C at seven-day intervals were investigated.

### Physicochemical analysis of yogurt fortified with nanoencapsulated fish oil

pH and acidity of yogurt were determined according to AACC (2003). To determine the amount of syneresis, 20 g of yogurt was centrifuged for 5 min at 28 g and the resulting supernatant volume was measured. The percentage of syneresis was performed according to Equation (2). (Ghorbanzade, Jafari et al. 2017):

(2) $$syneresis=\frac{\;separated\;liquid\;weight\;(g)}{\;yogurt\;\;total\;weight\;(g)}\times 100\;$$

### Fatty acid analysis data using GC-FID

Yogurt fat was first extracted with a mixture of chloroform: methanol (v/v (2: 1). Then, fatty acids were converted to methyl esters of fatty acids. In order to analyze fatty methyl esters, a gas chromatography (Luna, Juárez, & De la Fuente, 2005) was equipped with a 30 m long silica cap column with a diameter of 0.22 mm and a film thickness of 0.25 μm was used. The initial temperature was 158° C and increased by 2° C to 210 °C per minute and was kept at this temperature for 20 min. The injection valve temperature and the detector temperature were 230 °C and 240 °C, respectively. The carrier gas flow rate (helium) was 1.2 mL/min. GC injection was also performed by split method (Sharma and Ramanathan 2021).

### Determination of peroxide value (PV)

The peroxide value was detected according to the AOCS (2007). Briefly, the oil sample (3 g) was dissolved in glacial acetic acid (30 mL) and chloroform (20 mL) (3:2 v/v). Then saturated KI solution (1 mL) was added. The mixture was kept in the dark for 1 min, after adding of distilled water (50 mL), mixture was titrated against sodium thiosulfate (0.01 N). The PV value (mEq of oxygen/kg) was calculated using the following Equation (3):

(3) $$\mathrm{PV}\;value=\;1000\;\left(S\times N\right)\;\times W$$

where S is the volume of sodium thiosulfate solution (blank corrected) in ml, N is the normality of sodium thiosulfate solution and W is the weight of oil sample (g) (AOCS, 2007).

### Sensory evaluation

Twenty people (male and female) with an average age of 25–35 years were selected as panelists. Sensory evaluation was designed using a hedonic test based on scoring methods (Scale: 1-dislike extremely; 2-dislike slightly; 3-neither like nor dislike; 4-like slightly; 5-like extremely). In this method, each panelist was given a sample container with numbered three-digit codes, a spoon, and a glass of water along with a scoring form. In this way, each panelist evaluated characteristics such as taste and aroma, color, texture and overall acceptance for all samples (Ghorbanzade, Jafari et al., 2017).

### Statistical analysis

The tests were performed based on a completely randomized factorial design with three replications. One-way analysis of variance (ANOVA) and Duncan's mean comparison test at 5 % level were used to analyze the data. SPSS 25 statistical software was used in data analysis.

## Results and discussion

### Encapsulation efficiency of fish oil within BCD nanocomplexes

The encapsulation efficiency shows the ability of the wall material to preserve the core material and the durability of the core material by the wall and is one of the most important indicators of encapsulation evaluation. According to Jafari (Jafari, 2019a, Jafari, 2019b), many factors affect encapsulation efficiency, including the type of wall material or carrier material, core material or internal phase, wall-to-core ratio, encapsulation method, and so on. The results indicated that the EE (amount of encapsulated oil) within BCDs was 64 %. In our previous study, the efficiency of nanoliposomes containing fish oil was about 75 %. One of the reasons for the low EE of fish oil in BCDs compared to the nanoliposomal method could be smaller size of carriers (BCD). Jafari et al. (Jafari, 2019a, Jafari, 2019b) stated that a wall material alone cannot provide the required properties in nanoencapsulation, and the combination of two wall materials plays a better protective role. Similar observations have been reported by other authors in the literature (Ho et al., 2017, da Rocha Neto et al., 2018, Rakmai et al., 2018, Guo, 2019), it has been revealed that the EE is relatively low when BCD alone was used. On the other hand, some researchers have declared high EE of BCD when combined with other wall materials. Erfani et al. (2019) encapsulated cinnamon essential oil into nanoemulsions stabilized with BCD and sodium caseinate; they found that when BCD was used alone to encapsulate cinnamon essential oil, the EE was 70 %, while in the case of encapsulation with BCD-sodium caseinate, the EE increased significantly to 87 % (Erfani, Pirouzifard, Almasi, & Gheybi, 2019).

### Particle size of BCDs loaded with fish oil

**Fig. 1:**
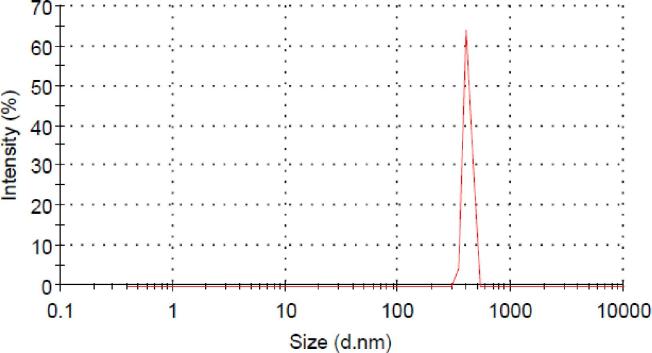
Size distribution of nanoencapsulated fish oil by β-cyclodextrin at room temperature and pH = 7.0.

Particle size measurement is one of the most important characteristic techniques for determining and confirming the production of nanoparticles. The size distribution results of nanoencapsulated fish oil revealed that the particle size range was estimated from 10 nm to below 1000 nm (Fig. 1). Furthermore, produced nanocarriers had a narrow particle-size range (mostly between 300 and 500 nm). The mean diameter of particles as well as polydispersity index (PDI) which represents the size distribution and uniformity of the system were 409 ± 1.22 nm and 0.557 ± 0.01, respectively. PDI is an indicator of the uniformity and dispersion of particles in the suspension system; the closer the number is to zero, the greater the uniformity of the particles. Therefore, based on the results, the obtained particles are homogeneous and uniform. The size of the nanoparticles obtained is close to previous reports (Chen et al., 2020, Jiang et al., 2021). Moreover, studies emphasize that there is an inverse relationship between particle size and EE so that with increasing particle size, EE decreases. Mazloom Hashemiravan Farhadyar & Farhadyar (2012) stated that the use of BCD alone to encapsulate essential oils of blueberry and raspberry resulted in the production of the smallest droplets due to nanoparticle structure; while in combination with inulin, the particle diameter increased. This rise in particle diameter is due to the sticking of particles together or changing the spatial structure of the wall material, which eventually led to a decrease in EE.

### Physicochemical properties of yogurt containing fish oil-loaded BCDs

#### pH and acidity

**Fig. 2:**
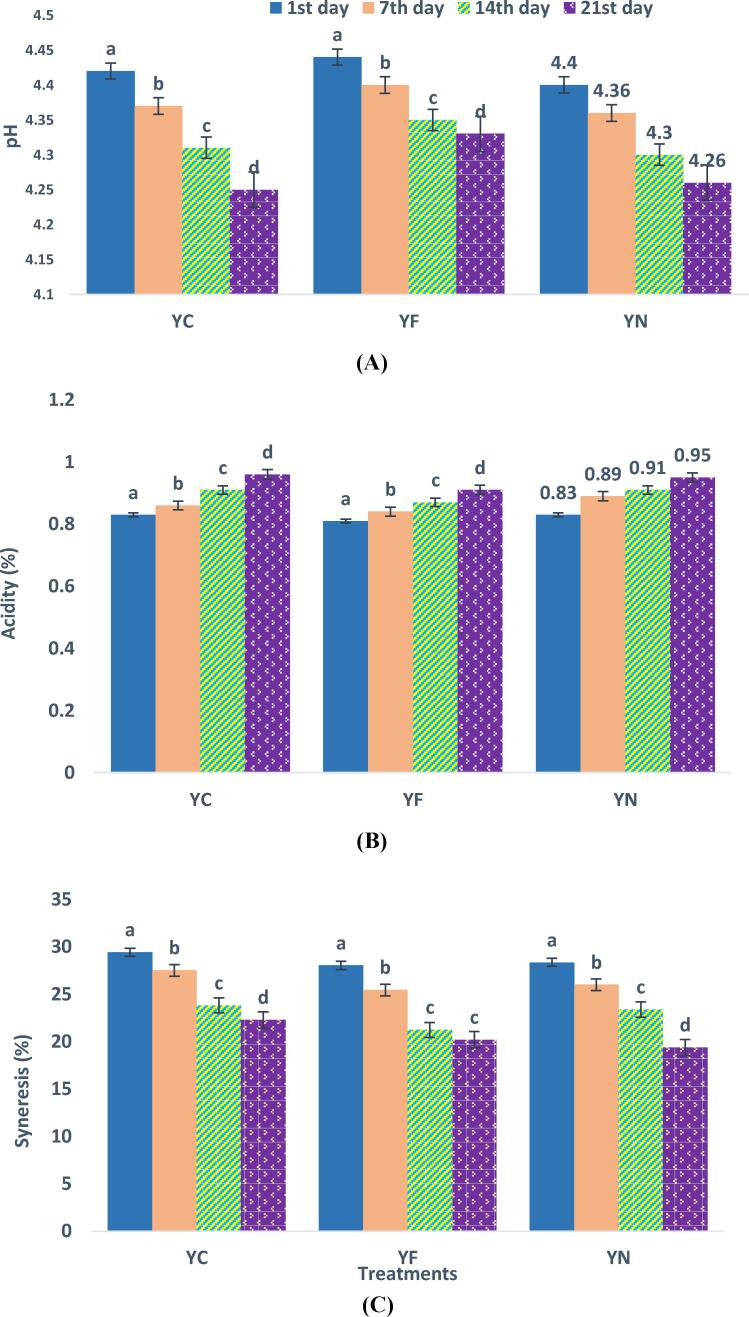
Variations in **(A)** pH, **(B)** acidity, and **(C)** syneresis of different yogurt samples during 21-day storage. YC: control sample (yogurt); YF: yogurt contains free form of fish oil; YN: yogurt contains nanoencapsulated fish oil by β-cyclodextrins.

The changes in pH and acidity of yogurt samples after 21 days of storage at 4 °C were investigated. The results showed that the initial pH in all samples of yogurt was about 4.43; pH decreased significantly (P < 0.05) in all samples over time due to the conversion of lactose to lactic acid by the starter culture. As can be seen in Fig. 2A, the control sample has the greatest pH reduction, whereas the yogurt containing free-form fish oil has the lowest pH reduction. Also, the results obtained from the measurement of acidity according to Fig. 2B, report an increasing trend of acidity in control yogurt more than other samples. Considering the inverse relationship of pH and acidity, it can be seen from the results that the incorporation of fish oil in the structure of yogurt slows down the process of decreasing pH and increasing acidity. The result also was in agreement with Zakipour Rahimabadi (2021) who reported a higher acidity of cheese fortified by encapsulated fish oil.

#### Syneresis

Synergy is one of the major quality defects in yogurt, which is actually called the appearance of whey on the surface of the yogurt. Evaluation of syneresis changes in all samples during storage at 4 °C is shown in Fig. 2C; the highest amount of syneresis occurred in both control and enriched samples during the first days of storage. This is due to the increase in acidity and fermentation during incubation and subsequent contraction of the gel network because of cooling, which leads to higher syneresis in the first days. However, over time in the following days and weeks, the amount of syneresis in all samples decreased due to the effect of lower pH on casein micelles and thus reducing the amount of serum release. Another point that can be noted is that the reduction of syneresis over time in samples enriched with fish oil was slightly greater than in the control sample. It can be said that in enriched samples, due to the increase in the amount of fat and, of course, the increase in total dry matter, the water holding capacity and the stability of the gel network increase and as a result, syneresis decreases. Gumus and Gharibzahedi (2021) in the evaluation of yogurts supplemented with lipid emulsions rich in omega-3 fatty acids, reported that syneresis in the yogurt fortification with microcapsules and emulsion systems entrapping omega-3 fatty acids is significantly lower than those yogurt samples containing free oil.

#### Fatty acid profile of yogurt containing nanoencapsulated fish oil

**Fig. 3:**
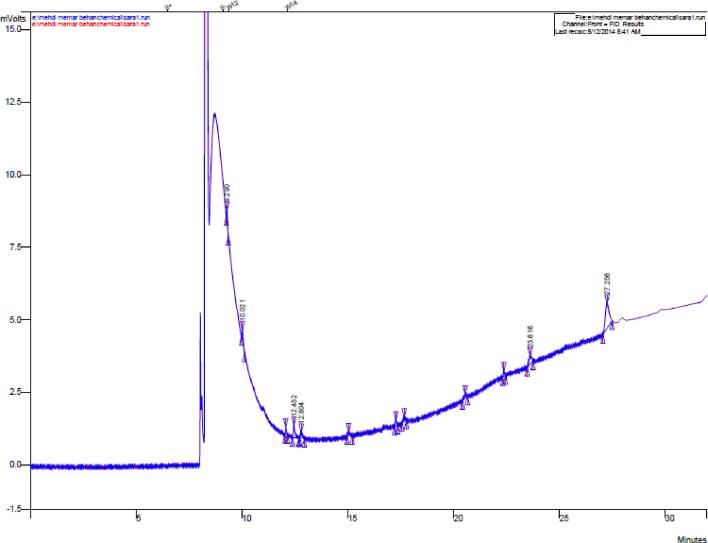
Chromatogram of fatty acids extracted from yogurt containing fish oil-loaded BCDs after storage (21 days) at 4 °C.

**Fig. 4:**
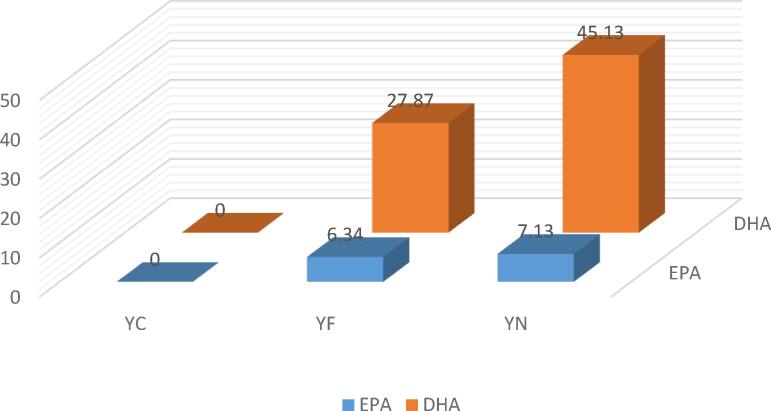
Amounts of EPA and DHA in yogurt samples after storage (21 days) at 4 °C. YC: control sample (yogurt); YF: yogurt contains free form of fish oil; YN: yogurt contains nanoencapsulated fish oil by β-cyclodextrins.

Chromatograms and profiles of fatty acids obtained from different yogurt samples after 21 days of storage at 4 °C, showed that fortification of yogurt with fish oil has a significant effect on increasing the amount of omega-3 fatty acids (DHA + EPA) (Fig. 3). According to Fig. 4, the highest amount of DHA and EPA remaining in the samples after 21 days is related to yogurt containing fish oil-loaded BCDs (45 % and 7 %, respectively). This is due to the presence of large amounts of these two fatty acids in fish oil. The difference in the amount of omega-3 fatty acids remaining in the sample of yogurts containing nanoencapsulated fish oil compared with those sample containing free fish oil indicates that the encapsulation process is efficient in preserving unsaturated fatty acids. Also, the addition of liposomes containing fish oil to yogurt showed higher levels of EPA and DHA after 21 days of storage in encapsulated samples. The difference in the amount of these two fatty acids in the two encapsulation methods is due to the greater stability and efficiency of liposomal systems than other methods (Ghorbanzade, Jafari et al., 2017).

#### Peroxide value

**Fig. 5:**
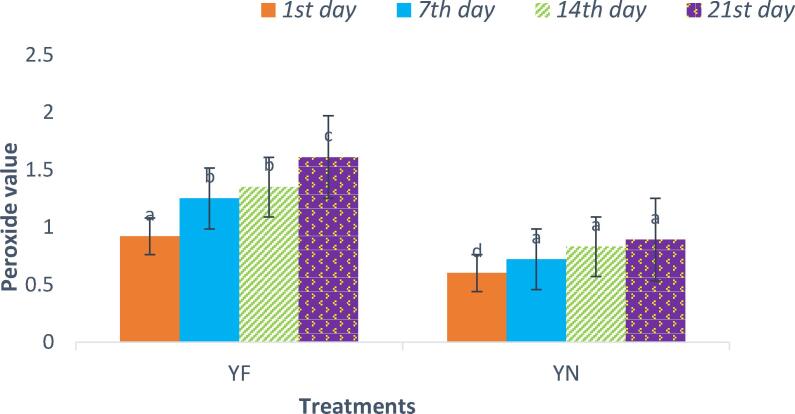
Peroxide value changes of different yogurt samples fortified with fish oil during 21 days storage. YF: yogurt contains free form of fish oil; YN: yogurt contains nanoencapsulated fish oil by β-cyclodextrins.

Peroxide is the primary product of the oxidation of fatty acids; in general, the higher the degree of unsaturation of oils, the higher their susceptibility to oxidation. When the amount of peroxide reaches a certain level, various changes take place in oils and fats, and aldehydes and ketones and short-chain fatty acids (second and third products) are created, which are effective in creating an unpleasant odor (Turek, Domagała, & Wszołek, 2018). There were significant differences regarding peroxide value (PV) in yogurt fortified with free fish oil and yogurt fortified with encapsulated fish oil by BCDs (P < 0.05). As shown in Fig. 5, the PV of oil extracted from yogurt containing the free form of fish oil increased from 0.92 (meq/kg) on the first day to 1.61 (meq/kg) on the 21st day, while the PV of yogurt containing encapsulated fish oil increased from 0.60 to 0.89 (meq/kg). The significant difference in the amount of PV in the control yogurt sample compared to the fortified samples is definitely related to the effectiveness of encapsulation by BCDs. The encapsulation protects the unsaturated fatty acids from destructive oxidation factors. Zhong et al. (2018) by adding fish oil in free and encapsulated forms to yogurt, found that PV in the yogurt containing encapsulated fish oil, after 21 days of storage, was significantly lower than the yogurt containing free from of oil.

#### Sensory evaluation

The sensory characteristics of yogurt samples such as texture, color, taste and aroma were evaluated by 20 panelists on the seventh day of storage (Table 1). The results indicated that the samples containing free fish oil received the lowest score in terms of all sensory characteristics, while the highest scores in terms of color and texture were related to the control sample as well as encapsulated fish oil. Regarding taste and aroma and overall acceptance, the panelists assigned the highest score to the control sample, which is mainly related to the specific taste of fish oil in yogurt compared to the control sample. All in all, according to data gathered from sensory evaluation in this study, adding nanoencapsulated fish oil into yogurt led to slight sensory differences compared to the control sample without fish oil.

**Table 1:** Sensory characteristics of the yogurt containing nanoencapsulated fish oil after 21 days storage.

Treatments	Sensory quality score			
	Taste and aroma	Color	Texture	Overall acceptance
YC	4.12 ± 0.04^a^	4.79 ± 0.05^a^	4–58 ± 1.05^a^	4.69 ± 1.08^a^
YF	3.79 ± 0.33^b^	2.60 ± 1.11^b^	3.74 ± 1.09^b^	2.71 ± 0.83^c^
YN	4.05 ± 0.12^a^	4.20 ± 0.02^a^	4.63 ± 0.09^a^	4.11 ± 0.22^b^

Values in the same column bearing different letters are significantly different.

YC: control sample (yogurt); YF: yogurt contains free form of fish oil; YN: yogurt contains nanoencapsulated fish oil by β-cyclodextrin.

## Conclusion

The results of this study found that BCDs were able to encapsulate and protect fish oil. The properties of encapsulated powders including particle size and efficiency indicated the successful effect of encapsulation. The use of powder encapsulated in yogurt also improved the properties of yogurt and less oxidation of fish oil compared to the free form, which all indicate the successful enrichment of yogurt with fish oil. Further studies should be performed to evaluate other properties of yogurt as well as controlled release and modeling of release to investigate the overall effect of fish oil encapsulation on yogurt.

## CRediT authorship contribution statement

**Tahere Ghorbanzade:** Data curation, Investigation, Methodology. **Sahar Akhavan-Mahdavi:** Formal analysis, Investigation, Methodology. **Mohammad Saeed Kharazmi:** Resources, Supervision, Validation, Visualization, Writing – review & editing. **Salam A. Ibrahim:** Resources, Validation, Visualization, Writing – review & editing. **Seid Mahdi Jafari:** Conceptualization, Project administration, Resources, Supervision, Validation, Visualization, Writing – review & editing.

## Declaration of Competing Interest

The authors declare that they have no known competing financial interests or personal relationships that could have appeared to influence the work reported in this paper.
